# Serum Paraoxonase-1 (PON-1) in Dogs: Frequency of Decreased Values in Clinical Practice and Prognostic Significance

**DOI:** 10.3390/vetsci12111066

**Published:** 2025-11-06

**Authors:** Virginia Bettoni, Filippo Tagliasacchi, Donatella Scavone, Alberto Galizzi, Chiara Locatelli, Maria Amati, Roberta Ferrari, Paola Scarpa, Saverio Paltrinieri

**Affiliations:** Department of Veterinary Medicine and Animal Sciences, University of Milan, 26900 Lodi, Italy; virginia.bettoni@unimi.it (V.B.); donatella.scavone@unimi.it (D.S.); alberto.galizzi2@gmail.com (A.G.); chiara.locaelli@unimi.it (C.L.); mariaamati80@gmail.com (M.A.); roberta.ferrari@unimi.it (R.F.); paola.scarpa@unimi.it (P.S.); saverio.paltrinieri@unimi.it (S.P.)

**Keywords:** paraoxonase-1 (PON-1), biomarker, acute phase protein, oxidative stress, hospitalization

## Abstract

Serum activity of paraoxonase-1 (PON-1) decreases in canine inflammation. This study aimed to evaluate how frequently PON-1 activity is reduced across different disease categories and to assess its potential prognostic significance. PON-1 activity was measured in 482 samples (435 first visit, 47 follow-up) collected during routine clinical activities. The emergency/first-opinion unit (EF) had the highest frequency of low values (18.3%) and lower median PON-1 activity (170.3 U/mL) compared with other units. The proportion of lower values and median PON-1 values were, respectively, significantly higher and lower in acute/severe diseases compared with chronic/mild diseases (13.3 vs. 7.1%; 177.0 vs. 192.9 U/mL) and in hospitalized compared with non-hospitalized dogs (27.7 vs. 4.5%; 150.0 vs. 193.0 U/mL). Low PON-1 activity may predict the need for hospitalization: values < 45.7 U/mL have a likelihood ratio of 14.4. The proportion of lower values and the median Paraoxonase-1 activity did not differ between survivors and non-survivors (18.2 vs. 25.0%; 162.0 vs. 161.3 U/mL). However, PON-1 activity diminished during hospitalization only in non-survivors. PON-1 activity should be measured in routine practice, especially in the EF; hospitalization may be warranted when results are markedly low. Decreases in PON-1 activity during hospitalization may suggest a negative outcome.

## Simple Summary

Paraoxonase-1 is a serum enzyme with antioxidant and anti-inflammatory properties, synthesized in the liver and associated with high-density lipoproteins. Its activity tends to decrease during systemic inflammation or oxidative stress, making it useful as a potential biomarker for disease severity and prognosis. This study analyzed the serum activity of paraoxonase-1 in 482 samples, evaluating its role in different hospital departments and its possible prognostic value. Results showed that the emergency/first-aid unit had the highest frequency of low PON-1 values and the lowest median. There were no significant differences between survivors and non-survivors in terms of mean PON-1 activity, but a reduction was observed during hospitalization in non-survivors. In conclusion, measurement of PON-1 is recommended in clinical practice, especially at admission to the emergency department, because very low values may indicate the need for hospitalization, and a further decline during hospitalization could indicate a poor prognosis.

## 1. Introduction

Paraoxonases (PONs) constitute a family of three enzymes, PON-1, PON-2, and PON-3, known for their antioxidant properties. Among these, paraoxonase-1 (PON-1) is the best characterized and was the first to be identified. This enzyme is a serum protein described in several species, including both invertebrates and mammals. Primarily synthesized in the liver and secreted into the bloodstream, PON-1 circulates bound to high-density lipoprotein (HDL) particles [1].

PON-1 is a calcium-dependent enzyme that exerts its antioxidant function mainly through arylesterase activity, hydrolyzing oxidized lipids and thereby protecting lipoproteins from oxidative modification [2,3,4,5]. In both human and animal studies, a marked decline in serum PON-1 concentration and enzymatic activity have been reported during the acute phase response. This reduction parallels the diminished activity of other HDL-associated molecules, collectively leading to a decreased systemic antioxidant capacity.

During inflammation, the composition of HDL undergoes profound structural remodeling. Levels of apolipoprotein A1, esterified cholesterol, and HDL-bound enzymes such as PON-1 are reduced, whereas acute-phase proteins (e.g., serum amyloid A and ceruloplasmin) are incorporated into the HDL particle [6]. Consequently, serum PON-1 activity decreases both due to reduced hepatic synthesis and to these compositional alterations, further compromising HDL’s antioxidative function [7].

In human medicine, reduced PON-1 activity has been documented in several disorders associated with oxidative stress, including cardiovascular diseases, chronic kidney disease (CKD), diabetes mellitus, chronic liver failure, and pancreatitis [8,9,10].

Compared with other animal species, the dog has been the subject of the largest body of research on PON-1. Among canine diseases, leishmaniosis, a condition strongly linked to oxidative stress [11], has been most extensively investigated. Additional studies have explored PON-1 alterations in babesiosis, ehrlichiosis, parvoviral and non-specific enteritis, sepsis, pancreatitis, atopic dermatitis, cardiac diseases, and hypercortisolism [12,13,14,15,16,17,18,19,20].

Overall, PON-1 activity appears to decline primarily in the presence of severe inflammatory processes [6,21], and such reductions are often inversely correlated with disease severity in multiple clinical contexts [15,22,23]. However, information remains limited regarding PON-1 behavior in non-infectious inflammatory diseases and its possible prognostic relevance in these conditions. 

Therefore, the present study aimed to assess, under routine veterinary practice conditions, the diagnostic and prognostic potential of PON-1 as a biomarker across various clinical presentations encountered in a veterinary hospital. These included cases characterized by primary inflammation, inflammation secondary to other underlying disorders (e.g., neoplasia, endocrinopathies), and/or oxidative stress. Specifically, by measuring PON-1 activity in samples randomly collected during routine clinical activity at our institution, we sought to determine in which hospital wards alterations in PON-1 activity occur most frequently and to what extent. Furthermore, we aimed to evaluate whether, irrespective of the underlying disease or reason for admission, reduced PON-1 activity was associated with increased illness severity, prolonged hospitalization, or death.

## 2. Materials and Methods

### 2.1. Animals and Sample Collection

Blood samples included in this study were prospectively collected during routine clinical activities at the Veterinary Teaching Hospital (VTH) from dogs admitted for diagnostic purposes or health check visits between April and September 2021. After completion of the requested diagnostic procedures, leftover serum samples were made available for research use.

Inclusion criteria comprised the availability of a sufficient volume of residual serum and the presence, in the hospital database, of relevant clinical information, including clinical presentation, presumptive or final diagnosis, short-term follow-up, and outcome, as detailed below. Informed consent for the possible research use of leftover biological material was obtained from all owners. Therefore, no additional approval from the Institutional Animal Care and Use Committee was required, in accordance with decision n° 2/2016 of the Ethical Committee of the University of Milan.

Blood was collected from each dog for diagnostic purposes after a 12 h fasting period, by venipuncture of the jugular, cephalic, or saphenous vein. Samples were drawn into potassium ethylenediaminetetraacetic acid (K_2_EDTA) tubes for complete blood count (CBC) analysis using an automated hematology analyzer (Sysmex XN-V, Sysmex Corporation, Kobe, Japan), and into plain tubes for serum biochemical testing.

Serum was separated within 15 min of collection, and biochemical parameters requested by the attending clinicians were measured using an automated chemistry analyzer (BT 3500, Biotecnica Instruments S.p.A., Rome, Italy) and reagents supplied by Futurlab S.r.l. (Limena, PD, Italy). Residual serum samples were subsequently aliquoted and immediately stored at −20 °C until analysis. Serum paraoxonase-1 (PON-1) activity was measured on the remaining sample volume using the same analyzer, as described below. Samples exhibiting hemolysis, lipemia, or icterus were excluded from the study to avoid analytical interferences.

When clinically indicated for patient management, follow-up samples were obtained during routine clinical care to monitor disease progression until discharge, death, or euthanasia. Residual serum from these follow-up samples was also included in the study, provided that the available volume was sufficient for PON-1 activity assessment.

Clinical information, including diagnostic findings and results of ancillary procedures available in the hospital database, was recorded for all dogs to enable subsequent classification into diagnostic categories, as described in the following sections. Additional data retrieved from the database, when available, included:

(1) Immediate follow-up information, used to stratify dogs into two groups—hospitalized vs. non-hospitalized.
(2) Outcome data, used to classify cases as survivors or non-survivors.

### 2.2. Measurement of PON-1 Activity

PON-1 activity was measured using a paraoxon-based method previously validated in several animal species, including cats, horses, cattle, and dogs [1,24,25,26]. In particular, the paraoxon-based method was already validated in dogs by Rossi et al. [6], who also determined also the reference interval (106.6–197.2 U/mL). Briefly, serum PON1 activity was measured spectrophotometrically using the automated chemistry analyzer BT 3500, Biotecnica Instruments SPA, Rome, Italy) using 6 mL of samples, that were incubated at 37 °C with 89 mL of distilled water and 100 mL of reaction buffer (glycine buffer 0.05 mM, pH 10.5 containing 1 mM of paraoxon-ethyl, purity > 90%, and 1 mM of CaCl_2_). The rate of hydrolysis of paraoxon to p-nitrophenol was measured by monitoring the increase in absorbance at 405 nm using a molar extinction coefficient of 18.050 L mol^−1^ cm^−1^. The unit of PON1 activity expressed as U/mL is defined as 1 nmol of p-nitrophenol formed per minute under the assay conditions.

### 2.3. Statistical Analysis

Statistical analyses were performed using Microsoft Excel and dedicated statistical software Analyse-it v.5.66 (Analyse-it Software Ltd., Leeds, UK). For all tests, statistical significance was set at *p* < 0.05.

Samples were grouped according to the hospital ward that submitted them, under the assumption that dogs with similar disease categories were managed within the same ward. The following wards were considered: cardiology, surgery, emergency/first opinion, diagnostic imaging, internal medicine, neurology, obstetrics and reproduction, and oncology. Results obtained from different wards were compared using a non-parametric analysis of variance for independent samples (Kruskal–Wallis test), followed by pairwise comparisons with the Mann–Whitney U test as a post hoc procedure. These analyses were performed considering only samples collected at the initial visit.

Further comparisons were carried out using the Mann–Whitney U test according to the following dichotomizations:

- Disease severity: dogs with acute or severe disease (e.g., life-threatening or septic conditions, acute infections, pulmonary edema, or end-stage neoplasia with systemic involvement) versus those with chronic or mild disease (e.g., non-traumatic orthopedic disorders, neoplasia without systemic complications), or dogs included in the absence of clinical signs (e.g., health checks, screening visits);
- Hospitalization status: hospitalized versus non-hospitalized dogs following initial evaluation;
- Outcome: survivors (improved clinical condition and/or discharge) versus non-survivors (spontaneous death or euthanasia related to the clinical episode), irrespective of hospitalization status.

Within the non-survivor group, an additional comparison was performed between dogs that died spontaneously and those euthanized, using a Kruskal–Wallis test followed by Mann–Whitney U post hoc analysis. All outcome-related analyses were also repeated considering only the subset of hospitalized dogs.

In hospitalized dogs with available follow-up samples, PON-1 activity at admission (T0) was compared with that measured at the second sampling (T1, within 15 days of T0), and subsequently with the last available measurement (T last), regardless of the time elapsed. These paired comparisons were performed separately for survivors and non-survivors using the Wilcoxon signed-rank test.

To evaluate the discriminative ability of PON-1 activity in predicting hospitalization, diagnostic performance indices were calculated for each operating point (i.e., each PON-1 value observed in the study population). The number of true positives, false positives, false negatives, and true negatives was determined as follows:

- True positives: hospitalized dogs with PON-1 values below the operating point;
- False positives: non-hospitalized dogs with PON-1 values below the operating point;
- False negatives: hospitalized dogs with PON-1 values above the operating point;
- True negatives: non-hospitalized dogs with PON-1 values above the operating point.

For each operating point, sensitivity (Sens), specificity (Spec), and positive likelihood ratio (LR^+^) were calculated using standard formulas [27]. A receiver operating characteristic (ROC) curve was then constructed by plotting Sens versus (1–Spec), and the area under the curve (AUC) was computed. Based on the ROC curve, the PON-1 value corresponding to the highest Youden index was identified, and its sensitivity and specificity were determined [27].

## 3. Results

### 3.1. Study Population

The study was performed on 482 samples (435 collected at first visit and 47 repeated samplings) from 401 dogs. Dogs with multiple hospitalizations for distinct clinical conditions were treated as independent cases and, when warranted by their clinical presentation, were reclassified into a different disease category. Mixed-breed dogs represented more than 27% of the population (specific data about prevalence of each breed are reported in Table 1).

Male dogs were prevalent (*n* = 160), followed by neutered males (*n* = 42), females (*n* = 102), and neutered females (*n* = 90). The gender was unspecified in seven dogs.

Based on clinical information reported at admission, ward of sampling and the final diagnosis formulated by the veterinarian that submitted the blood sample, dogs were grouped as reported in Table 2.

Information about the age of the sampled dogs was available in 380 cases. The median age of these 380 dogs was 8 years (age range 1 month–17 years). The comparison of age ranges recorded in the different groups revealed a significant difference (*p* = 0.001). The median age of dogs from the obstetric and reproduction ward was significantly lower than the median age of all the other groups (*p* < 0.001 vs. oncology; *p* < 0.01 vs. all the other groups), while the median age of oncology and cardiology ward were in general higher compared to that of the other groups considered. Specifically, statistically significantly higher median age was found in oncology compared to diagnostic imaging (*p* = 0.006), internal medicine (*p* = 0.040) and surgery (*p* = 0.016) patients and in cardiology compared to surgery (*p* = 0.045) and diagnostic imaging (*p* = 0.032) patients.

### 3.2. PON-1 Activity in the Different Groups

Data regarding PON-1 activity recorded in the different wards is reported in Table 3. Overall, 37 out of the 435 samples collected at the first visit (8.5%) had low PON-1 values. The emergency/first-opinion unit showed significantly lower PON-1 values compared with all other units, and the highest number and frequency of values lower than the reference interval. The surgery unit had significantly higher values than the internal medicine unit.

**Figure 1:**
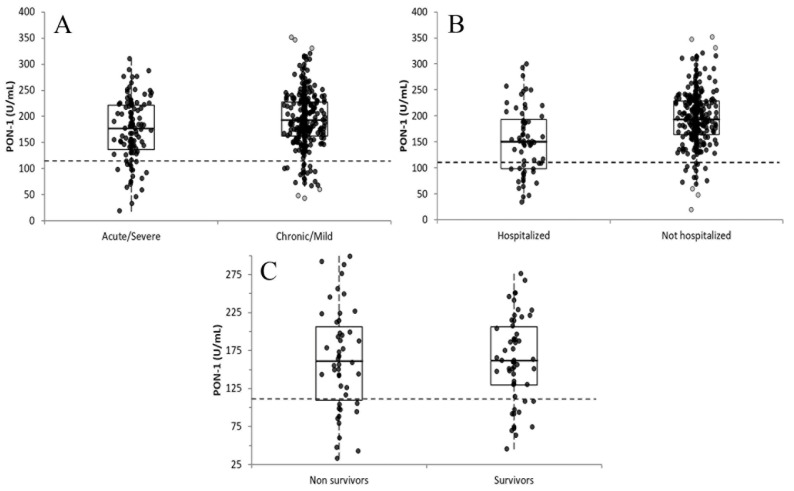
Distribution of results of dogs grouped in severe/acute clinical signs vs. chronic/mild clinical signs (**A**), hospitalized vs. non hospitalized (**B**) and survivors vs. non-survivors (**C**). In each graph, the boxes indicate the I–III interquartile range (IQR); horizontal lines indicate the median value. Vertical lines extend until the last value not classifiable as an outlier. Black circles indicate the results not classifiable as outliers; gray circles indicate the near outliers (values higher than the III quartile + 1.5 × IQR). The horizontal dashed line indicates the lower reference limit for PON-1 activity.

For 409/435 samples collected at admission, data on disease severity were available and allowed us to classify the dogs according to the clinical presentation: PON-1 activity was significantly lower in dogs with acute or severe disease (mean ± SD, 173.9 ± 59.6 U/mL; median, 177.0 U/mL; I–III interquartile range, 136.7–221.3 U/mL; *n* = 113) compared with dogs with chronic or mild disease (193.9 ± 53.1 U/mL; 192.9 U/mL; 162.6–228.0 U/mL; *n* = 296) (*p* = 0.003, Figure 1A). Moreover, the proportion of dogs with PON-1 activity lower than the lower reference limit was significantly higher (*p* = 0.049) in the acute/severe group (15/133 cases, 13.3%) than in the chronic/mild group (21/296 cases, 7.1%).

**Figure 2:**
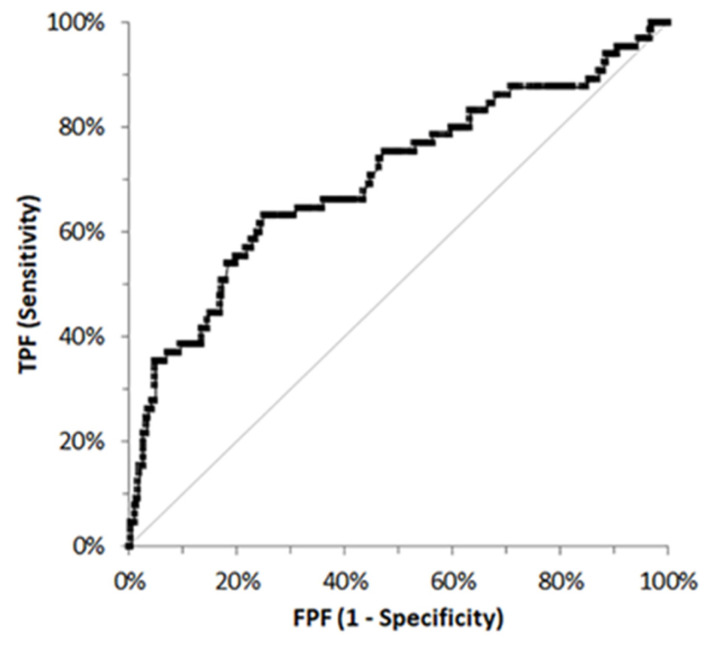
Receiver operating characteristic curve built using the values of PON-1 activity in hospitalized and non-hospitalized dogs. The central gray line represents the no-discrimination line.

In 378 cases information about hospitalization or discharge of dogs was available. PON-1 activity was significantly lower in hospitalized dogs (152.6 ± 64.1 U/mL; 150.0 U/mL; 97.9–192.6 U/mL; *n* = 65) compared with non-hospitalized dogs (195.5 ± 51.4 U/mL; 193.0 U/mL; 163.7–228.3 U/mL; *n* = 313) (*p* < 0.001, Figure 1B). Moreover, the proportion of dogs with PON-1 activity lower than the lower reference limit was significantly higher (*p* < 0.001) in hospitalized dogs (18/65 cases, 27.7%) than in non-hospitalized dogs (14/313 cases, 4.5%). The ROC curve analysis designed to assess the discriminating power of PON-1 in detecting dogs requiring hospitalization, showed an AUC (0.702; 95% CI = 0.624–0.780) significantly different from the line of no discrimination (*p* < 0.001, Figure 2). The highest Youden index (0.382) corresponded to 164.00 U/mL (specificity: 75.1%; sensitivity: 63.1%), which is not clinically relevant since it is within reference interval. However, using the lower limit of the reference interval as a threshold to predict the need for hospitalization, the specificity was 95.8%, the sensitivity was 27.7% and the LR+ was 6.67. The highest LR+ (14.4) and absolute specificity were reached at cut-offs of 45.7 U/mL and 32.8 U/mL, respectively.

For 103 cases, information regarding the outcome was available. No significant differences (*p* = 0.994) were observed between survivors (163.0 ± 56.8 U/mL; 162.0 U/mL; 130.0–206.4 U/mL; *n* = 55) and non-survivors (163.2 ± 66.8 U/mL; 161.3 U/mL; 109.6–206.5 U/mL; *n* = 48, Figure 1C), even when considering dogs that spontaneously died (158.4 ± 84.6 U/mL; 156.0 U/mL; 86.6 ± 213.6 U/mL; *n* = 19) and dogs subjected to euthanasia (166.4 ± 53.6 U/mL; 167.2 U/mL; 136.8–196.3 U/mL; *n* = 29) as separate groups (*p* = 0.189). The proportion of dogs with PON-1 activity lower than the lower reference limit did not significantly differ (*p* = 0.399) between survivors (10/55 cases, 18.2%) and non-survivors (12/48 cases, 25.0%).

The same trend was observed when restricting the analysis to 47 samples collected at admission from 40 hospitalized dogs. Significant differences were not observed (*p* = 0.965) between survivors (178.4 ± 56.0 U/mL; 173.0 U/mL; 141.0–218.3 U/mL; *n* = 30) and non-survivors (182.0 ± 76.2 U/mL; 175.0 U/mL; 116.9–247.2 U/mL; *n* = 17), even when considering dogs that died spontaneous (169.7 ± 52.1 U/mL; 156.4 U/mL; 155.2–192.0 U/mL; *n* = 7) and dogs subjected to euthanasia (184.6 ± 60.54 U/mL; 191.0 U/mL; 136.2–221.5 U/mL; *n* = 10) as separate groups (*p* = 0.875). The proportion of dogs with PON-1 activity lower than the lower reference limit did not significantly differ (*p* = 0.650) between survivors (5/30 cases, 16.7%) and non-survivors (2/17 cases, 11.8%).

### 3.3. PON-1 Activity During the Follow Up

Forty-seven samples collected during the follow-up were available for analysis. The number of sequential samples varied according to the clinical presentation. In 12 cases, only 1 follow-up sample was available, 9 from dogs that were discharged after hospitalization and 3 from dogs that died or were euthanized. In 9 dogs multiple follow-up samples were available (2 in 3 cases, 3 in 2 cases, 4 in 2 cases, 6 in one case, 9 in one case), Moreover, the schedule of sequential samplings was influenced by the initial clinical presentation, the course of the disease, and the time of occurrence of either death or discharge.

A univocal trend was not observed among dogs with different outcomes (Table 4).

**Figure 3:**
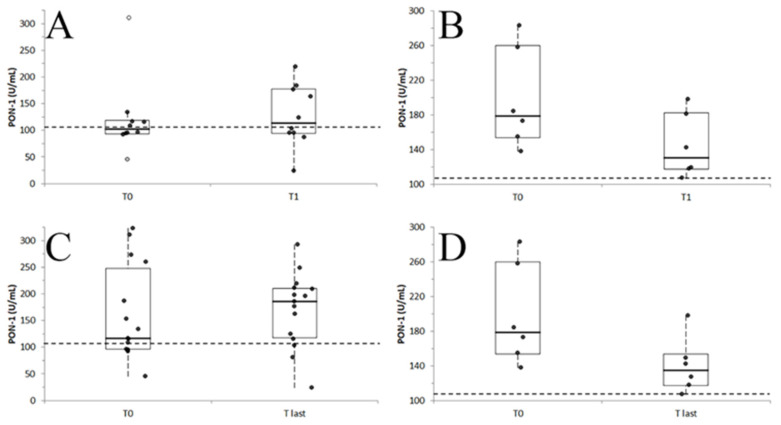
Distribution of results recorded at T0 or in the second sampling (T1) in dogs that survived (**A**) or died (**B**) and of results recorded at T0 (**C**) or in the last sampling (T last) in dogs that survived (**C)** or died (**D**); white circle indicate the far outliers (values higher than the III quartile + 3.0 × IQR). In each graph, the boxes indicate the I–III interquartile range (IQR); horizontal lines indicate the median value. Vertical lines extend until the last value not classifiable as an outlier. Black circles indicate the results not classifiable as outliers; gray circle indicate the near outliers (values higher than the III quartile + 1.5 × IQR). The horizontal dashed line indicates the lower reference limit for PON-1 activity.

In 16 dogs for which a follow-up sample was available within 15 days from admission (mean interval: 3.8 ± 4.1 days; median: 2 days), PON-1 concentrations at admission (T0) were compared to those at the time of the second sampling (T1) (Figure 3A,B). In dogs with a negative outcome (*n* = 6), a statistically significant decrease in PON-1 activity was observed (*p* = 0.031), with values dropping from 198.5 ± 58.5 U/mL at T0 (median: 178.5 U/mL; range: 153.6–260.1 U/mL) to 144.2 ± 37.3 U/mL at T1 (median: 130.5 U/mL; range: 117.2–182.4 U/mL). In contrast, no significant difference was found in dogs with a positive outcome (*n* = 10; *p* = 0.679), with PON-1 levels of 121.0 ± 70.7 U/mL (median: 102.4 U/mL; range: 93.6–311.0 U/mL) at T0 and 127.2 ± 58.1 U/mL (median: 113.7 U/mL; range: 94.3–219.0 U/mL) at T1.

Similarly, when comparing the first (T0) and last sampling available (T last) regardless of the time interval between them (Figure 3C,D), significant differences were not observed (*p* = 0.679) for dogs that survived (T0: 160.4 ± 88.8 U/mL; 117.0 U/mL; 95.9–247.8 U/mL; T1: 169.9 ± 69.4 U/mL; 186.0 U/mL; 117.5–210.7 U/mL; *n* = 15). Conversely, in dogs with a negative outcome, PON-1 activity at the last sampling was significantly lower than at admission (*p* = 0.031), decreasing from 198.5 ± 58.5 U/mL at T0 (median: 178.5 U/mL; range: 153.6–260.1 U/mL) to 140.2 ± 32.2 U/mL at T last (median: 134.5 U/mL; range: 117.2–153.3 U/mL).

## 4. Discussion

In the present study, serum paraoxonase-1 (PON-1) activity was assessed in a large canine population admitted to a referral veterinary hospital. The findings indicated that decreased PON-1 activity occurred in approximately 8.5% of first-visit samples. The lowest PON-1 values and the highest proportion of sub-reference results were observed among dogs presented to the emergency and first-opinion unit. Moreover, animals affected by acute or severe diseases, as well as those requiring hospitalization, exhibited significantly reduced PON-1 activity compared with dogs suffering from chronic or mild conditions and those not hospitalized. Although PON-1 activity at admission did not reliably differentiate survivors from non-survivors, a progressive decline in PON-1 concentration during hospitalization tended to characterize non-surviving patients. Finally, while its discriminative ability for mortality was limited, markedly decreased PON-1 activity was associated with a substantially increased likelihood of hospitalization.

The finding that only a modest percentage of dogs (8.5%) exhibited PON-1 activity below the reference interval suggests that severely depressed PON-1 is not ubiquitous in routine clinical cases but nonetheless occurs with sufficient frequency to warrant attention. Based on its antioxidant properties, the serum activity of PON-1 has been reported to decrease in several conditions associated with oxidative stress [25,28,29]. Among these, metabolic diseases such as diabetes mellitus, cardiovascular diseases, and inflammation are the most studied, since they are all characterized by the release of oxidative compounds of different origin [30,31] that may lead to the consumption of antioxidant molecules such as PON-1. Moreover, inflammation is a reported cause of a decrease in hepatic production of PON-1, classified as a negative acute phase protein [32]. Additionally, the main site of PON-1 production is the liver and therefore decreased PON-1 synthesis may occur when liver disease or dysfunction is present [33,34,35,36]. However, no information on the possible prognostic role of decreased serum PON-1 activity, which has been demonstrated in people, has so far been provided in dogs [37,38,39].

The emergency/first-opinion unit recorded the lowest PON-1 levels and the highest prevalence of sub-reference values, consistent with the fact that this unit treats a higher proportion of dogs with acute or life-threatening diseases. These conditions are often accompanied by oxidative stress and systemic inflammation, which have been shown to reduce PON-1 activity in both veterinary and human medicine [21,22,40]. In contrast, other hospital units (e.g., internal medicine, oncology) also displayed reduced PON-1 values, albeit to a lesser extent. This likely reflects the presence of chronic inflammatory or oxidative processes (for example in metabolic disorders or neoplasia) within these patient populations [14,15,22,41,42]. Meanwhile, the relatively higher PON-1 levels observed in the surgery unit may reflect the predominance of elective or chronic-condition surgeries (e.g., orthopedic, neoplastic) rather than acute, systemic illness.

The significantly lower PON-1 activity in dogs with acute/severe disease compared with those with chronic/mild conditions supports the hypothesis that PON-1 is negatively impacted by systemic inflammation and oxidative stress. The greater proportion of sub-reference values in the acute/severe group further underscores its potential as a biomarker of disease severity. Moreover, dogs requiring hospitalization exhibited lower PON-1 values and a higher prevalence of sub-reference results than non-hospitalized dogs. Although the receiver-operating characteristic (ROC) analysis indicated limited standalone diagnostic utility for predicting hospitalization, the positive likelihood ratio (LR^+^) analysis revealed that dogs with PON-1 values below the reference interval were approximately seven times more likely to require hospitalization. This likelihood increased more than fourteen-fold for values below 45.7 U/L and became absolute at values below 32.8 U/L. These findings suggest that severely reduced PON-1 activity may heighten the clinician’s suspicion of systemic disease warranting inpatient care.

In contrast to its apparent value for assessing disease severity or hospitalization need, PON-1 activity at admission did not discriminate between survivors and non-survivors, even when distinguishing spontaneous deaths from euthanasia. Also, the absence of consistent trends in PON-1 changes during the follow-up, suggest that PON-1 alone is not a reliable predictor of mortality. This lack of prognostic discrimination may reflect the wide dispersion of PON-1 values within both outcome groups, many of which remained within the reference interval, irrespective of eventual prognosis. Additionally, acute severe conditions with initially low PON-1 may recover with treatment, while chronic conditions with poor outcomes may present with normal PON-1 values. However, in dogs with negative outcomes, a progressive decline in PON-1 activity was observed during hospitalization. This suggests that serial monitoring of PON-1 may provide incremental prognostic information: specifically, failure of PON-1 to recover or a downward trend may mark a failing physiological response or worsening disease. In fact, although PON-1 levels at admission did not differentiate survivors from non-survivors, our findings suggest that non-survivors can be identified by monitoring PON-1 activity during hospitalization: specifically, if PON-1 levels remain low or decrease over time rather than increasing.

Our results support the potential utility of PON-1 activity as a general marker of systemic illness severity in dogs, rather than a disease-specific marker. While PON-1 should not be considered a standalone prognostic tool for mortality based on current evidence, its measurement may aid in the early identification of patients at higher risk of requiring hospitalization and in monitoring response to therapy through serial sampling. Several limitations must be acknowledged. First, the overlapping of disease types across hospital units (for example, neoplasia treated in multiple wards) and the heterogeneous patient population (varied pathophysiological mechanisms including metabolic, inflammatory, and degenerative conditions) restrict disease-specific interpretation. Future applications of this study could involve classifying patients based on organ or systems. Second, breed and age heterogeneity across groups may introduce confounding, although the large caseload mitigates this risk. The age of dogs from the reproduction ward was lower than in other groups, likely due to the multitude of pre-surgical panels that young patients undergo before programmed ovariectomies and orchiectomies. Conversely, patients of the oncology ward tended to have a higher median age compared to the other wards. Despite some differences in the median age, no neonatal or very young dogs were included, an important consideration since such patients may exhibit inherently lower PON-1 activity [24,43,44,45]. Third, uncontrolled variables, including prior or ongoing medications, disease duration, and variable follow-up intervals, could have influenced PON-1 values. Specifically, follow-up samples were collected at variable time intervals dictated by clinical need rather than a standardized protocol, limiting the ability to perform uniform paired analyses. Finally, by enrolling a heterogeneous hospital population, this study lacks detailed etiological analysis, preventing the assessment of how specific pathophysiological mechanisms (e.g., inflammation vs. oxidative stress vs. hepatic dysfunction) contribute to PON-1 alterations. Nonetheless, these limitations reflect the real-world nature of a veterinary hospital setting and do not invalidate, in our opinion, the potential clinical relevance of PON-1 measurement.

## 5. Conclusions

PON-1 activity was significantly lower in dogs with acute or severe disease compared with dogs affected by chronic or mild disease. Moreover, hospitalization, regardless of the ward, is also associated with a statistically significant reduction in the biomarker’s activity, proving that PON-1 could be a useful marker of disease severity and that hospitalization might be required when admission values are particularly low.

However, its utility as a predictive marker is limited because it does not differentiate survivors from non-survivors. Serial measurements may be more informative than a single value at admission, particularly for monitoring disease progression in critically ill dogs. In this context, it should be emphasized that sample timing and follow-up intervals were inconsistent, which limited the robustness of longitudinal interpretations of PON-1 dynamics and hindered a clear understanding of temporal trends.

A potential clinical application of these findings could be the measurement of PON-1 activity at hospital admission, particularly in dogs presented to emergency units, and possibly, though to a lesser extent, in those admitted to internal medicine or oncology wards. Low PON-1 activity values, especially those below the reference interval, might warrant closer clinical attention and a more cautious approach to hospitalization decisions.

Furthermore, serial measurement of PON-1 activity during hospitalization or follow-up may offer additional information regarding disease progression. The absence of an early increase in PON-1 activity after admission could potentially indicate a poorer prognosis. However, these hypotheses warrant further validation in larger, prospective studies before PON-1 testing can be implemented as a routine clinical tool.

Finally, measurement of PON-1 activity may be recommended as a screening test, since low values may indicate a more severe condition that may require hospitalization. In the authors’ view, prospective studies employing multi-biomarker approaches and focusing on specific diseases or patient stratification by organ or system involvement are warranted to validate the prognostic and clinical utility of PON-1 in veterinary medicine. Future research should explore how PON-1 interacts with inflammation, oxidative stress, and specific disease processes and progression to better understand its clinical relevance.

## Figures and Tables

**Table 1:** Prevalence of the different breeds included.

Breed	*n*
Mixed breed	109
Labrador Retriever	20
French Bouledogue	18
Poodle	16
Golden Retriever	15
German Shepherd, Jack Russell Terrier	13
Bernese Mountain Dog	11
Boxer, NR	10
Cocker Spaniel, Dachshund	9
Rottweiler	8
Dobermann, Maltese, Weimaraner	7
Chihuahua, CKCS	6
Maremmano-abruzzese Sheepdog, Staffordshire American Terrier	5
Border Collie, Dogo Argentino, Epagneul Breton, Fox terrier, German Shorthaired Pointer, Pit bull, Shih Tzu, WHWT	4
Akita Inu, Beagle, Bracco Italiano, English Setter, Pug, Siberian Husky, Yorkshire Terrier	3
Australian Shepherd, Belgian Shepherd, Bolognese, Boston Terrier, Bull Terrier, Dalmatian, English Bulldog, Greyhound, Miniature Poodle, Rhodesian Ridgeback, Whippet	2
Afghan Hound, American Bulldog, Bobtail, Caucasian Shepherd, Cane Corso, Czechoslovakian Wolfdog, Dogue de Bordeaux, Entlebucher Mountain Dog, Flat Coated Retriever, Foxhound, German Wirehaired Pointer, Italian Greyhound, Italian Hound, Leonberger, Newfoundland, Pinscher, Pointer, Samoyed, Shar Pei, Shetland Sheepdog, Schnauzer, Spitz, Springer Spaniel	1

CKCS = Cavalier King Charles Spaniel; NR = not reported; WHWT = West Highland White Terrier.

**Table 2:** Summary of the information recorded in the hospital database regarding the diagnosis or the procedures reported for the dogs included in the different groups.

Ward	Type of Disease/Procedure
**Cardiology** **(*n* = 55)**	**MVD** [***n* = 28**: mild (ACVIM B1) (*n* = 11), moderate (ACVIM B2) (*n* = 2), severe (ACVIM C) (*n* = 11), or associated with other valvular dysfunctions or conditions such as neoplasia (*n* = 1), CKD (*n* = 1) edema (*n* = 3)]; **control** [***n* = 15**: breed screening (*n* = 8), wellness visits (*n* = 7)]; **miscellaneous** [***n* = 9**: pulmonary hypertension, PDA, atrial rupture, cardiac murmur, pulmonary stenosis, cardiac tumor, ventricular tachycardia, cardiac tamponade, pulmonary thromboembolism (*n* = 1 each)]; **Addison disease** [***n* = 2**]
**Diagnostic imaging (*n* = 39)**	**Neoplastic masses** [***n* = 15**: carcinoma (*n* = 4), mast cell tumor (*n* = 3), splenic masses (*n* = 2), osteosarcoma (*n* = 2), meningioma, abdominal mass not further investigated, adrenal mass, sarcoma (*n* = 1 each)]; **osteo-muscular symptoms** [***n* = 11**: Back pain (*n* = 6), lameness (*n* = 4), CMO (*n* = 1)]; **neurological symptoms** [***n* = 5**: Chiari-like syndrome (*n* = 2), discal hernia (*n* = 2), intracranic mass (*n* = 1)]; **abdominal abnormalities** [***n* = 4**: splenomegaly, likely inflammatory origin (2), focal enteropathy (*n* = 1), ureteral dysplasia (*n* = 1)]; **controls** [***n* = 4**: Screening for breed diseases (*n* = 4)]
**Emergency/first opinion (*n* = 93)**	**Inflammation** [***n* = 29**: respiratory symptoms (coughing, discharge, rhinitis, *n* = 9), cystitis (*n* = 4), fever responsive to anti-inflammatory treatments (*n* = 3) pyometra (*n* = 3), bacteriemia (*n* = 3), otitis (*n* = 3), ab ingestis pneumonia (*n* = 1) cutaneous abscess (*n* = 1), parvovirus (*n* = 1), peritonitis (*n* = 1)]; **gastrointestinal signs** [***n* = 20**: Vomiting and/or diarrhea with acute clinical presentation]; **foreign bodies** [***n* = 11**: cutaneous (*n* = 5), gastrointestinal (*n* = 4), nasal (*n* = 2)]; **chronic or acute bleeding** [***n* = 5**: ulcerated tumors (*n* = 2), splenic hematoma (*n* = 1), hemoperitoneum (*n* = 1), ear wound (*n* = 1)]; polytrauma [***n* = 5**: car accident (*n* = 5)]: **acute urinary disorders** [***n* = 4**: urinary obstruction (*n* = 3), AKI (*n* = 1)]; **checkup visit** [***n* = 4**]; **dilatation/volvulus** [***n* = 4**: gastric volvulus dilatation (*n* = 3), intestinal intussusception (*n* = 1)]; **cachexia in neoplastic patients** [***n* = 4**: unclassified abdominal tumor (*n* = 1), lymphoma (*n* = 1) mast cell tumor (*n* = 1), carcinoma (*n* = 1)]; neurological symptoms [***n* = 3**: acute paraplegia (*n* = 2), convulsions (*n* = 1)]; **rodenticide poisoning** [***n* = 2**]; **portosystemic shunt** [***n* = 2**]
**Internal medicine (*n* = 80)**	**CKD** [***n* = 24**]; **dermatological signs** [***n* = 10**: pruritus and generalized dermatitis (*n* = 5), interdigital dermatitis (*n* = 3), nodular pyogranulomatous panniculitis (*n* = 1) sarcoptic and otodectic mange (*n* = 1)]; **gastrointestinal diseases** [***n* = 8**: lymphoplasmocytic gastritis or enteritis (*n* = 3), hemorrhagic enteritis (*n* = 2), giardiasis (*n* = 2), malabsorption (*n* = 1)]; **leishmaniasis** [***n* = 8**]; endocrine diseases [***n* = 6**: diabetes mellitus (*n* = 3), hypothyroidism (*n* = 2), hyperadrenocorticism (*n* = 1)]; wellness visit [***n* = 6**]; chronic hepatopathy [***n* = 4**]; **immune-mediated polyarthritis** [***n* = 4**]; **urinary disorders** [***n* = 4**: urolithiasis (*n* = 3) recurrent cystitis (*n* = 1)]; **hematological disorders** [***n* = 3**: immune-mediated thrombocytopenia (*n* = 2), immune-mediated hemolytic anemia (*n* = 1)]; **miscellaneous** [***n* = 3**: cataract (*n* = 1), dirofiraliasis (*n* = 1), splenic sarcoma (*n* = 1)]
**Neurology (*n* = 17)**	**idiopathic epilepsy** [***n* = 5**]; **peripheral neuritis** [***n* = 5**]; **ataxia** [***n* = 2**]; **vestibular syndrome** [***n* = 2**]; **prosencephalic syndrome** [***n* = 1**]; **discal hernia** [***n* = 1**]; **paraplegia** [***n* = 1**]
**Obstetric/** **reproduction (*n* = 25)**	**castration** [***n* = 7**]; **gynecological checkup** [***n* = 5**]; **pregnancy monitoring** [***n* = 4**]; **mammary neoplasm** [***n* = 3**]; **cystic endometrial hyperplasia** [***n* = 2**]; **ovariectomy** [***n* = 2**]; **congenital abnormality** [***n* = 1**]; **dystocia** [***n* = 1**]
**Oncology (*n* = 84)**	**mast cell tumor** [***n* = 22**]; **carcinoma** [***n* = 20**]; **lymphoma** [***n* = 17**]; **sarcoma** [***n* = 11**: **soft tissue sarcoma** (*n* = 6); perivascular wall tumor (*n* = 3); hemangiosarcoma (*n* = 1), histiocytic sarcoma (***n*** = 1)]; miscellaneous [***n* = 6**: thymoma, tricoblastoma, intraarticular tumor, leiomyosarcoma, sertolioma, retromandibular tumor (*n* = 1 each)]; **hepatic tumor** [***n* = 3**]; **melanoma** [***n* = 3**]; **plasma cell tumor** [***n* = 2**]
**Surgery** **(*n* = 42)**	**orthopedic** [***n* = 12**]; **neoplasia** [***n* = 11**]; **BOAS** [***n* = 4**]; **dental surgery** [***n* = 3**]; **chronic otitis** [***n* = 3**]; **miscellaneous** [***n* = 3**: bronchocscopy (***n*** = 1), syalocele (***n*** = 1), urethral surgery (***n* = 1**)] **perineal hernia** [***n* = 2**]; **laryngeal surgery** [***n* = 2**]; **nasal surgery** [***n* = 2**];

AKI = acute kidney injury; BOAS = Brachycephalic Obstructive Airway Syndrome; CKD = chronic kidney disease; CMO = cranio-mandibular osteopathy; MVD = mitral valve disease; PDA = patent ductus arteriosus. In bold the title of subgrups of conditions included in the caseload of each hospital unit.

**Table 3:** PON-1 values recorded in the different groups of dogs. Data regarding PON-1 activity in U/mL are reported as mean ± standard deviation, median (between parentheses), I–II interquartile and (between parenthesis) min–max values.

Ward	*n*	PON-1 (U/mL)	Low PON-1
**Cardiology (CA)**	55	199.9 ± 56.8 (202.4) **^EF^ 165.2–237.0 (74.3–346.0)	4 (7.3%)
**Diagnostic Imaging (DI)**	39	192.4 ± 51.4 (189.3) *^EF^ 155.6–232.7 (47.2–292.0)	2 (5.1%)
**Emergency/first opinion (EF)**	93	166.3 ± 58.3 (170.3) ***^OR,SU;^ **^CA,NE,ON;^ *^DI,IM^ 131.1–205.3 (32.8–315.0)	17 (18.3%)
**Internal medicine (IM)**	80	185.5 ± 61.3 (184.0) *^SU,EF^ 144.8–225.3 (18.5–351.0)	7 (8.8%)
**Neurology (NE)**	17	211.1 ± 52.7 (228.3) **^EF^ 152.8–247.0 (130.0–287.4)	0 (0.0%)
**Obstetric/reproduction (OR)**	25	205.0 ± 32.8 (202.0) ***^EF^ 179.3–229.9 (136.0–262.0)	0 (0.0%)
**Oncology (ON)**	84	191.5 ± 52.4 (189.6) **^EF^ 156.5–223.6 (66.2–314.0)	5 (6.0%)
**Surgery (SU)**	42	203.4 ± 39.6 (203.0) ***^EF;^ *^IM^ 183.6–234.2 (80.7–290.0)	2 (4.8%)
		*p* = 0.002	*p* = 0.015

* *p* < 0.05; ** *p* < 0.01; *** *p* < 0.001. The superscripts refer to the different groups being compared, and that the meaning of each superscript abbreviation is provided after the name of each group in column 1.

**Table 4:** Recorded PON-1 values over time in the samples from dogs repeatedly sampled during the follow up. T0 corresponds to the basal sampling and T1 to T9 are the sequential sampling collected after T0 Data regarding PON-1 activity are reported as U/mL. Results lower than the lower limit of the reference interval are reported in bold.

Outcome	T0	T1	T2	T3	T4	T5	T6	T7	T8	T9
Discharge	311.0	219.0								
Discharge	323.0	249.0								
Discharge	273.0	293.0								
Death	258.0	181.0	225.0	237.0	149.2					
Discharge	117.0	124.0	180.0	199.0	186.0					
Discharge	187.0	125.0								
Death	184.0	107.0								
Discharge	108.0	184.0	120.0	198.0						
Death	155.0	142.0								
Discharge	134.0	**95.3**	**81.6**	**88.6**	**103.0**	**101.0**	166.0	124.0	197.0	196.0
Discharge	**91.8**	**94.9**	**106.0**	110.0	183.0	228.0	211.0			
Euthanasia	283.0	198.0	220.0							
Euthanasia	173.0	119.0	149.0	127.0						
Discharge	**45.7**	**23.8**								
Discharge	**96.7**	**87.2**	**80.7**							
Discharge	153.0	116.0								
Discharge	**95.7**	177								
Discharge	115.9	163.1	162							
Euthanasia	138	118.1								
Discharge	**93.8**	**103.5**								
Discharge	260	209								

## Data Availability

The original contributions presented in this study are included in the article. Further inquiries can be directed to the corresponding author.

## Abbreviations

The following abbreviations are used in this manuscript:

AKI	Acute kidney injury
BOAS	Brachycephalic Obstructive Airway Syndrome
CKD	Chronic kidney disease
CKCS	Cavalier King Charles Spaniel
CMO	Cranio-mandibular osteopathy
HDL	High density lipoprotein
LDL	Low density lipoprotein
MVD	Mitral valve disease
PDA	Patent ductus arteriosus
PON	Paraoxonase
PON-1	Paraoxonase-1
SAA	Serum amyloid A
VTH	Veterinary teaching hospital
WHWT	West Highland White Terrier
